# Non-Criteria Antiphospholipid Antibodies in Women with Recurrent Pregnancy Loss

**DOI:** 10.3390/biomedicines14061177

**Published:** 2026-05-22

**Authors:** Madina Khalmirzaeva, Gulfiruz Urazbayeva, Almagul Kurmanova, Nagima Mamedalieva, Gaukhar Kurmanova, Damilya Salimbayeva, Ainur Veliyeva, Gaini Anartayeva, Zhanar Kypshakbayeva, Shugyla Amirtayeva, Altynay Nurmakova

**Affiliations:** 1 Faculty of Medicine and Healthcare, Al-Farabi Kazakh National University, Almaty 050040, Kazakhstan; madinakhalmirzaeva7@gmail.com (M.K.); mamedalieva_kz@mail.ru (N.M.); gaukhar.kurmanova@kaznu.kz (G.K.); ainura.veliyeva@gmail.com (A.V.); ecomed_gaini@mail.ru (G.A.); kypshakbaevazhanar@gmail.com (Z.K.); amirtayeva@gmail.com (S.A.); 2 Department of Strategic Development and Science, Scientific Center for Obstetrics, Gynecology and Perinatology, Almaty 050010, Kazakhstan; sdamilya@mail.ru (D.S.); nur_altinay01@mail.ru (A.N.)

**Keywords:** recurrent pregnancy loss, non-criteria antiphospholipid antibodies, immunoblot, risk stratification, annexin V, β_2_-glycoprotein I, prothrombin

## Abstract

**Background**: Recurrent pregnancy loss (RPL) remains etiologically unexplained in 40–50% of cases following standard diagnostic workup. Non-criteria antiphospholipid antibodies (non-criteria aPL) are increasingly considered potential markers of seronegative obstetric antiphospholipid syndrome (APS); however, their diagnostic value in this clinical setting requires further investigation. **Objective**: To assess the diagnostic value of non-criteria aPL in women with RPL and to construct an exploratory immunological scoring model for diagnostic stratification. **Methods**: Antiphospholipid antibody detection was performed using a single-measurement semi-quantitative line immunoblot assay (Anti-Phospholipid 10 Dot, Generic Assays, Germany). Statistical analysis included χ^2^, Fisher’s exact test, Mann–Whitney U test, binary logistic regression, and ROC analysis. **Results**: Statistically significant associations with RPL were observed for anti-prothrombin antibodies (OR = 11.1; 95% CI 1.8–68.0; *p* = 0.022 [Haldane–Anscombe correction]), anti-annexin V (OR = 4.28; 95% CI 1.18–15.6; *p* = 0.023), and anti-β_2_GP I (OR = 3.31; 95% CI 1.18–9.28; *p* = 0.019). The exploratory composite immunological score demonstrated moderate discriminatory performance (AUC = 0.701; 95% CI 0.588–0.814; *p* = 0.005). The overall logistic regression model was statistically significant (χ^2^ = 8.564; *p* = 0.036), although none of the individual predictors retained independent significance, indicating a contribution of cumulative immunological burden rather than any single marker. **Conclusions**: In this single-center cross-sectional study, non-criteria aPL were frequently detected in women with RPL and were statistically associated with the condition. The findings should be interpreted as hypothesis-generating only, given the cross-sectional design, single-measurement immunoblot, small control group, and absence of external validation. Confirmation in larger prospective multicenter cohorts using ELISA-based assays with the internationally recommended 12-week repeat measurement is required before any clinical implementation.

## 1. Introduction

Recurrent pregnancy loss (RPL), defined as two or more spontaneous pregnancy losses, affects approximately 1–3% of women of reproductive age and represents one of the most clinically and psychologically challenging reproductive disorders [1,2,3]. According to the 2023 ESHRE guideline update, up to 40–50% of RPL cases remain etiologically unexplained, even after a comprehensive diagnostic workup, suggesting the existence of underrecognized pathogenetic mechanisms [1,2].

Among established immune-mediated causes of RPL, antiphospholipid syndrome (APS) occupies a central position. APS is a systemic autoimmune disease associated with a broad spectrum of adverse obstetric outcomes, including early and late pregnancy loss, placental insufficiency, and thrombotic complications [4,5]. The classical laboratory diagnosis of APS requires the detection of at least one antibody from three antibody groups—lupus anticoagulant, anticardiolipin antibodies, or anti-β_2_-glycoprotein I antibodies—with positivity confirmed on two separate occasions at least 12 weeks apart, as defined by the international classification criteria and EULAR recommendations [5,6]. Detection of any one of these markers is sufficient for classification; positivity for all three is not required.

However, evidence accumulated over the past decade indicates that the existing criteria have limited sensitivity, particularly in the obstetric setting [6,7,8]. A substantial proportion of patients with clinical manifestations of APS, including RPL, test negative on standard serology, leading to the concept of seronegative obstetric APS [9,10,11]. In this context, growing attention has been directed towards non-criteria antiphospholipid antibodies—antibodies to annexin V, prothrombin, the phosphatidylserine/prothrombin complex, phosphatidylethanolamine, phosphatidylinositol, and phosphatidic acid—which may be detected in patients with clinical APS features in the absence of classical markers and have been associated with adverse pregnancy outcomes [12,13,14,15,16,17,18].

Contemporary research is increasingly highlighting that the clinical relevance of non-criteria aPL is not uniform: the strongest evidence is for antibodies to annexin V, prothrombin, and extended β_2_GP I epitopes, while data on the other markers remain conflicting [13,17,18,19,20]. Furthermore, it has been suggested that the immune response due to the simultaneous presence of multiple antibodies, rather than isolated seropositivity, may play a key role in determining reproductive risk [18,19,21].

The updated 2023 ACR/EULAR classification criteria for APS, aimed at more precise risk stratification, still do not incorporate the broad spectrum of non-criteria antibodies [6], creating a clinical paradox in which patients with overt obstetric disease may remain outside formalized diagnostic categories. From a practical standpoint, the inclusion of non-criteria aPL in the diagnostic panel is considered a promising approach; however, unified interpretation algorithms have yet to be established [6,14,18].

Rationale and aim of the study: Despite the growing volume of literature on non-criteria aPL, robust observational data from Central Asian populations are lacking, and the relative diagnostic value of individual non-criteria specificities—together with their potential combination into a single integrated index—has not been characterized in this region. Furthermore, most existing studies use heterogeneous methodologies and target panels, which complicates comparative interpretation. The present study was therefore designed to (i) describe the prevalence and spectrum of non-criteria aPL in a single-center cohort of Kazakhstani women with RPL, (ii) quantify the strength of association between individual specificities and RPL, and (iii) explore—in a hypothesis-generating manner—whether a simple composite immunological score could provide additional diagnostic information beyond classical APS markers in this population.

## 2. Materials and Methods

### 2.1. Study Design and Participants

An analytical cross-sectional observational study was conducted. A total of 120 women of reproductive age were enrolled: a study group of 100 patients with recurrent pregnancy loss (≥2 spontaneous losses) and a control group of 20 women with a history of uncomplicated pregnancy and live-born children. The cross-sectional design does not allow inference of temporal sequence or causal relationships.

Exclusion criteria comprised the following: (i) chromosomal abnormalities in either partner confirmed via karyotyping or microarray analysis; (ii) clinically significant somatic comorbidities—namely, uncontrolled diabetes mellitus, decompensated thyroid disease, chronic kidney disease (eGFR < 60 mL/min/1.73 m^2^), chronic liver disease, malignancy, and known systemic autoimmune disease meeting classification criteria (e.g., systemic lupus erythematosus); (iii) confirmed congenital uterine malformations; (iv) acute infectious disease at the time of enrolment.

The minimum calculated sample size for the study group was 96 (Z = 1.96; expected proportion *p* = 0.5; absolute precision d = 0.1). The smaller control group (*n* = 20) was a pragmatic constraint of single-center recruitment of women with strictly uncomplicated obstetric histories; its implications for statistical power are addressed in the Limitations section.

### 2.2. Immunological Testing

#### 2.2.1. Test System and Specimen Collection

Antiphospholipid antibody detection was performed via semi-quantitative line immunoblot assay using the commercial test system Anti-Phospholipid 10 Dot (REF 5012; GA Generic Assays GmbH, Blankenfelde-Mahlow, Germany; IVD/CE-marked). The assay simultaneously detects IgG and IgM antibodies against ten phospholipid antigens and protein cofactors in human serum, cardiolipin, phosphatidic acid, phosphatidylcholine, phosphatidylethanolamine, phosphatidylglycerol, phosphatidylinositol, phosphatidylserine, annexin V, β_2_-glycoprotein I (β_2_GP I), and prothrombin, plus a positive control. Manufacturer-reported analytical performance: antiphospholipid IgG sensitivity 67%, specificity 94%; IgM sensitivity 91%, specificity 82%.

Fresh serum specimens were obtained by means of standard antecubital venepuncture. Lipemic, hemolyzed, or bacterially contaminated samples were excluded. Sera were stored at −20 °C until analysis; repeated freeze–thaw cycles were not permitted. All kit components were equilibrated to room temperature (18–25 °C) for at least 30 min before use.

#### 2.2.2. Procedure and Result Interpretation

Analyses were performed in incubation troughs on a horizontal orbital shaker according to the manufacturer’s protocol: serum incubation (30 µL undiluted serum, 30 min at room temperature with continuous shaking), washing, conjugate incubation (50 µL IgG or IgM conjugate, 15 min), washing, substrate incubation (0.5 mL 3,3′,5,5′-tetramethylbenzidine, 10–12 min), reaction termination, drying, and qualitative reading against the manufacturer’s interpretation template. A specimen was considered positive for a given antigen if the staining intensity exceeded that of the corresponding control line and negative if staining intensity was equal to or below the control line. The test validity criterion required an intensely stained positive control line. All procedures were performed in accordance with Good Laboratory Practice principles.

Single measurement and choice of assay format: Antibody testing was performed once at enrolment. The international classification criteria for APS require confirmation of positivity on two separate occasions at least 12 weeks apart; this was not performed in the present study, which is acknowledged as a methodological limitation. Furthermore, the line immunoblot platform—although IVD/CE-marked and convenient for simultaneous multi-antigen screening—is semi-quantitative, has lower standardization than ELISA, and has limited inter-laboratory comparability. Manufacturer-defined cut-offs were used; in-house cut-off validation was not performed. These features are explicitly considered in the Discussion and Limitations.

In addition to the immunoblot panel, all participants underwent assessment of classical APS criteria markers: lupus anticoagulant by means of the standard coagulometric two-step screening/confirmation procedure, anticardiolipin antibodies, and anti-β_2_GP I antibodies (the latter two also covered by the immunoblot panel).

### 2.3. Clinical Data Collection

A standardized history was taken for all participants, including obstetric–gynecological and somatic data. Endometrial thickness was recorded only as a baseline descriptive variable from the most recent transvaginal ultrasound performed in the early/mid-proliferative phase of the cycle (cycle days 5–9) within six months prior to enrolment; this variable was not used as an exposure or outcome in any inferential analysis. Data on intrauterine procedures were collected to characterize the cohort; these data are descriptive only and were not interpreted as risk factors for RPL, given their well-known association with prior pregnancy losses (i.e., reverse causality).

### 2.4. Statistical Analysis

Statistical analysis was performed using IBM SPSS Statistics (version 26.0; IBM Corp., Armonk, NY, USA). Categorical variables were compared using the χ^2^ test or Fisher’s exact test (when expected cell counts were small). Continuous variables are reported as median and interquartile range (Me [IQR]) and were compared using the Mann–Whitney U test. Association strength was quantified as odds ratios (OR) with 95% confidence intervals (CI). The Haldane–Anscombe correction (+0.5) was applied when zero-cell counts were present; the resulting OR estimates carry wide CIs and should be interpreted with caution. Independent predictors were identified by means of binary logistic regression; model fit was assessed using the Hosmer–Lemeshow test and Nagelkerke R^2^. The discriminatory performance of the exploratory immunological score was evaluated through ROC analysis (AUC with 95% CI). Statistical significance threshold: *p* < 0.05. No artificial intelligence-based statistical tools were used; the previously included statement in this regard has been retracted as inaccurate.

### 2.5. Ethical Approval

This study was approved by the Ethical Committee of Kazakhstan Medical University “Higher School of Public Health” (Code: IRB-70-2023, dated 6 May 2025). All participants provided written informed consent for the use of biomaterials in this study, in accordance with the Declaration of Helsinki.

## 3. Results

### 3.1. Reproductive History and Baseline Clinical Characteristics

A total of 120 women were enrolled: 100 (83.3%) in the study group and 20 (16.7%) in the control group. There were no missing values. The groups were comparable in age (32 [29–36] vs. 31 [28–35] years; *p* = 0.41) and BMI (23 [21–26] vs. 22 [20–24] kg/m^2^; *p* = 0.28). As expected, the study group demonstrated significantly more pregnancies, more pregnancy losses of all gestational ages, and a higher frequency of prior intrauterine procedures than the control group (Table 1).

Baseline endometrial thickness was thinner in the study group; this is reported descriptively and is not used in any subsequent inferential analysis.

### 3.2. Antiphospholipid Antibody Spectrum

Antibody frequency: On single-measurement immunoblot screening, at least one antiphospholipid antibody was detected in 79 (79.0%) of the 100 study-group patients. Among these 79 seropositive patients, the median number of distinct antibody specificities per patient was 1 (IQR: 1–2; range: 1–4): a single specificity was detected in 34 cases, two in 22 cases, three in 19 cases, and four in 4 cases. This distribution illustrates a tendency towards co-occurrence of multiple antibody specificities but should not be interpreted as evidence of a defined clinical APS phenotype since (i) single time-point detection does not satisfy the 12-week confirmation requirement of the international criteria, and (ii) several of the detected specificities are non-criteria markers.

**Figure 1:**
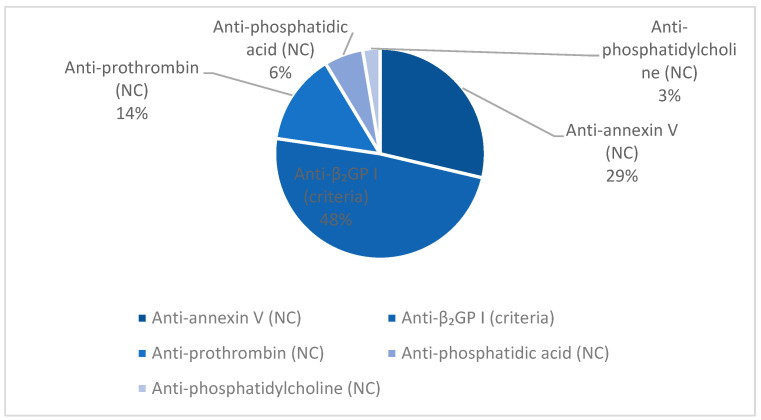
Distribution of antiphospholipid antibodies with recurrent pregnancy loss (RPL). NC—non-criteria specificity.

Antibody distribution: The most prevalent specificities were anti-β_2_GP I (73%), anti-annexin V (43%), and anti-prothrombin (21%) (Figure 1).

It should be emphasized that anti-β_2_GP I is included in the classical APS criteria; the high prevalence of anti-β_2_GP I therefore does not reflect a predominance of non-criteria over criteria specificities at the antibody level. Among non-criteria specificities, anti-annexin V was the most frequent.

Association with RPL: Statistically significant between-group differences were demonstrated for three non-criteria aPL (Table 2).

The strongest association was observed for anti-prothrombin antibodies (OR = 11.1; 95% CI: 1.8–68.0; *p* = 0.022), pointing to their potential role in disrupting coagulation homeostasis at the level of decidual vasculature and trophoblast. Anti-annexin V (OR = 4.28; 95% CI: 1.18–15.6; *p* = 0.023) and anti-β_2_GP I (OR = 3.31; 95% CI: 1.18–9.28; *p* = 0.019) showed moderate but consistent associations, which aligns with their established roles in anticoagulant dysregulation and endothelial function. No significant differences were found for the remaining markers, likely due to their low prevalence in the sample and the limited statistical power due to the small control group.

Methodological note: For anti-prothrombin, anti-phosphatidic acid, and anti-phosphatidylcholine antibodies, OR estimates were calculated using the Haldane–Anscombe correction due to the presence of zero cell counts in the control group; absolute OR values should therefore be interpreted with caution.

### 3.3. Multivariable Analysis

Binary logistic regression, using the three antibody specificities with significant univariate associations as predictors, yielded a statistically significant overall model (χ^2^ = 8.564; *p* = 0.036; Hosmer–Lemeshow *p* = 0.952; Nagelkerke R^2^ = 0.116). However, none of the individual predictors retained independent statistical significance:

- Anti-annexin V: OR = 3.07 (95% CI: 0.72–13.02; *p* = 0.129);
- Anti-β_2_GP I: OR = 2.07 (95% CI: 0.68–6.25; *p* = 0.198);
- Anti-prothrombin: OR not stably estimable due to zero cell count (excluded from the final model).

This is a globally significant model in which no single predictor reaches independent significance, indicating that the association with RPL is driven by the cumulative immunological burden of multiple co-occurring specificities rather than by any single antibody acting independently. The modest Nagelkerke R^2^ also reflects this distributed contribution.

### 3.4. Exploratory Composite Immunological Score

To explore—in a hypothesis-generating manner—whether co-occurrence of multiple specificities has diagnostic value, an unweighted composite immunological score was constructed from the three specificities with significant univariate associations (anti-annexin V, anti-β_2_GP I, and anti-prothrombin). Each marker was assigned a value of 0 (absent) or 1 (present), and the values were summed (range: 0–3).

Threshold-specific operating characteristics are summarized in Table 3.

Receiver operating characteristic (ROC).

**Figure 2:**
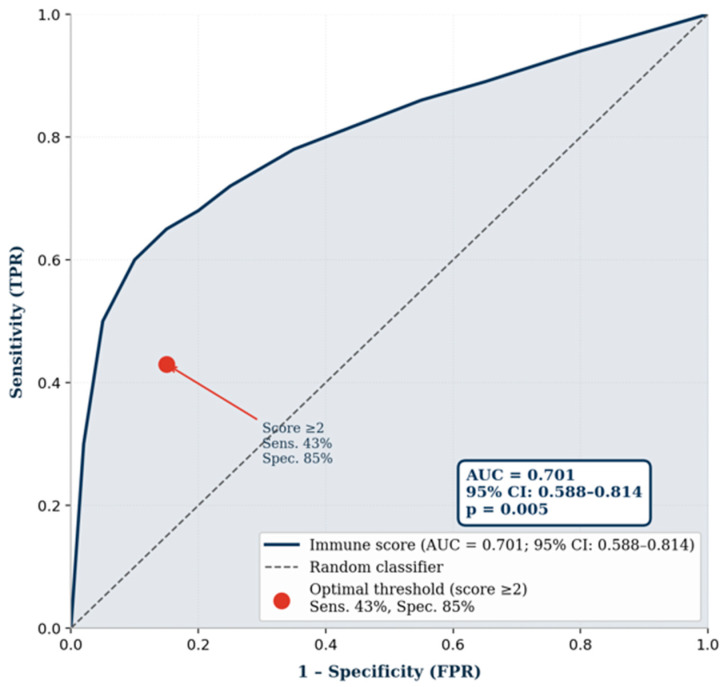
Receiver operating characteristic (ROC) curve for the exploratory composite immunological score (anti-annexin V + anti-β_2_GP I + anti-prothrombin) for distinguishing women with RPL from controls. AUC = 0.701; 95% CI 0.588–0.814.

ROC analysis demonstrated moderate discriminatory performance: AUC = 0.701 (95% CI 0.588–0.814; *p* = 0.005; Figure 2).

These data are presented for descriptive purposes only. The score was developed and tested on the same single-center cohort without independent internal split, k-fold cross-validation, or external validation; AUC values derived in this manner are subject to optimism bias and overfitting. The reported AUC of 0.701 is therefore best characterized as a preliminary, hypothesis-generating signal rather than as evidence of a validated diagnostic instrument.

## 4. Discussion

The present cross-sectional observational study describes the prevalence and spectrum of antiphospholipid antibodies—including non-criteria specificities—in a single-center cohort of women with recurrent pregnancy loss. Three observations merit particular attention: the high overall prevalence of seropositivity (79%), the broad polyspecificity of the immune response, and the contribution of cumulative immunological burden—rather than any single antibody—to the association with RPL [6,7,8,9,10,13].

Cumulative immunological burden rather than individual markers: Univariate analyses identified three specificities significantly associated with RPL: anti-prothrombin, anti-annexin V, and anti-β_2_GP I. However, in multivariable logistic regression—although the overall model was significant—none of these specificities retained independent significance. This pattern is most consistent with a contribution from the combined immunological profile rather than from any single antibody acting independently. The finding aligns with the concept articulated by Jara et al. [13] and Caraiola et al. [15] that cumulative burden, rather than isolated seropositivity, may be the more clinically meaningful construct in non-criteria/seronegative obstetric APS. Accordingly, our discussion of individual markers below should not be read as a causal claim about each antibody in isolation, but as a description of which specificities contributed most to the overall pattern.

Detection of non-criteria specificities: Anti-annexin V (43%), anti-prothrombin (21%), and lower-prevalence specificities (anti-phosphatidic acid, anti-phosphatidylcholine) were detected in a substantial proportion of the cohort. This is consistent with reports by Mercier et al. [7], Beça et al. [16,18], and Martínez-Taboada et al. [17], who describe a meaningful prevalence of non-criteria specificities in obstetric APS cohorts that fail to satisfy the formal classification criteria. Our data add a single-center Central Asian observation to this growing body of literature; they do not, in themselves, establish that these specificities are causally involved in pregnancy loss.

Mechanistic plausibility of the most prominent specificities: The strongest univariate association in our data was observed for anti-prothrombin antibodies. Such antibodies have been proposed to interfere with thromboregulation at the maternal–fetal interface, and similar associations have been described by Mu et al. [19] and Foddai et al. [8]. Anti-annexin V antibodies may attenuate the anticoagulant shield formed by annexin V on the trophoblast surface and placental endothelial cells [14,15]. Antibodies to β_2_GP I—which is also a criterion target—modulate β_2_GP I receptor activity, principally via interaction with domain I [6,15]. These mechanisms provide biological plausibility for the observed associations but do not demonstrate causality in our cross-sectional data.

Exploratory composite score: The composite immunological score showed an AUC of 0.701, which corresponds to moderate discriminatory performance. We deliberately characterize this as a preliminary, hypothesis-generating signal: the score was developed and tested on the same single-center cohort, without an independent internal split, cross-validation, or external validation; consequently, the AUC is subject to optimism bias and the threshold-specific sensitivity/specificity values should not be transferred to clinical decision-making in their present form. A comparable AUC was reported by Beça et al. (*n* = 91) [18], which provides external context but does not constitute validation of our specific score.

Methodological considerations: assay format and confounding: Antibody detection was performed via a single-measurement semi-quantitative line immunoblot. Although IVD/CE-marked and convenient for multi-antigen screening, this format has well-recognized limitations in comparison with quantitative ELISA: lower standardization, semi-quantitative readout dependent on visual line-intensity comparison, limited inter-laboratory comparability, and absence of locally validated cut-offs. Inter-assay variability was not formally assessed in the present work. In addition, the international classification criteria for APS require confirmation of antibody positivity at ≥12 weeks, which was not performed here. These constraints—combined with the small control group, the absence of multivariable adjustment for potential confounders such as prior intrauterine procedures, and the single-center setting—substantially limit the strength of inference that can be drawn.

Clinical context: 2023 ACR/EULAR criteria: The 2023 ACR/EULAR criteria raised the diagnostic threshold for classical APS, with implications for obstetric APS coverage [6,8,9]. Our data do not allow direct comparison of criteria fulfilment across cohorts; however, they support the broader observation that a meaningful subgroup of women with RPL exhibits non-criteria seropositivity, and they reinforce the call from the literature [14,16,22,23] for additional research evaluating whether expanded antibody panels, used with rigorous methodology, can usefully complement classical testing.

Limitations: Specific limitations of the study are as follows: (1) cross-sectional design, which precludes any causal or prognostic inference and disallows assessment of temporal sequence; (2) the small control group (*n* = 20), which inflates the variance of OR estimates, limits statistical power, and led to the use of Haldane–Anscombe correction for multiple comparisons (with the resulting wide confidence intervals); (3) single-time-point antibody measurement, which does not satisfy the international criteria for confirmation at ≥12 weeks; (4) use of a semi-quantitative line immunoblot rather than quantitative ELISA, with manufacturer-defined rather than locally validated cut-offs and unassessed inter-assay variability; (5) absence of internal cross-validation or external validation of the composite immunological score, with attendant risk of optimism bias; (6) absence of formal multivariable adjustment for potential confounders, including prior intrauterine procedures and infectious history; and (7) single-center design, limiting generalizability. These limitations should be addressed in larger prospective multicenter studies using ELISA-based assays and the internationally recommended repeat-measurement protocol.

## 5. Conclusions

In this single-center cross-sectional observational study, antiphospholipid antibodies—predominantly anti-β_2_GP I, anti-annexin V, and anti-prothrombin—were frequently detected in women with recurrent pregnancy loss. The associations between these specificities and RPL were statistically significant in univariate analyses; however, none retained independent significance in multivariable regression, suggesting that the observed signal reflects cumulative immunological burden rather than the effect of any individual antibody. An exploratory composite immunological score showed moderate discriminatory performance (AUC = 0.701) but should be regarded as preliminary and hypothesis-generating, given the absence of internal cross-validation or external replication.

These findings cannot be interpreted as evidence of causality, prognostic value, or readiness for clinical application. Confirmation in larger prospective multicenter cohorts using ELISA-based assays with the internationally recommended 12-week repeat-measurement protocol—together with formal validation of any composite score—is required before non-criteria aPL testing can be considered for incorporation into the diagnostic workup of women with otherwise unexplained RPL.

## 6. Patent

In connection with the present line of work, a Utility Model application has been filed (Patent for Utility Model No. 11926; application No. 2025/1777.2; 20 March 2026). The relevance of this filing for the interpretation of the present manuscript is addressed in the Conflicts of Interest statement below.

## Figures and Tables

**Table 1:** Baseline clinical and demographic characteristics.

Parameter	Study Group (*n* = 100)	Control Group (*n* = 20)	*p*
Age, years (Me [IQR])	32 (29–36)	31 (28–35)	0.41
BMI, kg/m^2^ (Me [IQR])	23 (21–26)	22 (20–24)	0.28
Number of pregnancies	5 (3–6)	2 (1–3)	<0.001
Number of deliveries	2 (1–3)	1 (0–2)	0.01
Spontaneous miscarriages <10 weeks	1 (0–2)	0 (0–0)	<0.001
Pregnancy losses >10 weeks	0 (0–1)	0 (0–0)	0.02
Missed abortion	2 (1–3)	0 (0–0)	<0.001
Endometrial thickness (M-echo), mm—descriptive only *	7.2 (5.8–8.8)	8.4 (7.5–9.2)	0.01
Prior intrauterine procedures, n (%)—descriptive only **	72 (72.0%)	0 (0%)	<0.001

Me [IQR]—median [interquartile range]; BMI—body mass index. * Endometrial thickness was measured by means of transvaginal ultrasound in the early/mid-proliferative phase (cycle days 5–9) within six months prior to enrolment and is reported descriptively only. ** Prior intrauterine procedures (uterine evacuation, hysteroscopy) are reported descriptively; given their well-established association with prior pregnancy losses (reverse causality), they are not interpreted as causal risk factors for RPL.

**Table 2:** Antiphospholipid antibodies and their association with recurrent pregnancy loss.

Marker	Study Group *n* (%)	Control *n* (%)	OR (95% CI)	*p*
Anti-annexin V (NC)	43 (43.0%)	3 (15.0%)	4.28 (1.18–15.6)	0.023
Anti-β_2_GP I (criteria)	73 (73.0%)	9 (45.0%)	3.31 (1.18–9.28)	0.019
Anti-prothrombin (NC)	21 (21.0%)	0 (0%)	11.1 (1.8–68.0) *	0.022
Anti-phosphatidic acid (NC)	9 (9.0%)	0 (0%)	4.26 *	0.353
Anti-phosphatidylcholine (NC)	4 (4.0%)	0 (0%)	1.91 *	1.000

NC—non-criteria specificity. * Estimated with Haldane–Anscombe correction (zero cell count in the control group); β_2_GP I—β_2_-glycoprotein I; OR—odds ratio; CI—confidence interval.

**Table 3:** Operating characteristics of the exploratory composite immunological score at different thresholds.

Threshold	Sensitivity	Specificity	Interpretation
Score ≥ 1	74%	55%	Higher sensitivity, lower specificity
Score ≥ 2	43%	85%	Balanced sensitivity and specificity
Score ≥ 3	20%	100%	Maximum specificity, very low sensitivity

## Data Availability

Clinical–pathological data are available.
